# Marker Development for Phylogenomics: The Case of Orobanchaceae, a Plant Family with Contrasting Nutritional Modes

**DOI:** 10.3389/fpls.2017.01973

**Published:** 2017-11-21

**Authors:** Xi Li, Baohai Hao, Da Pan, Gerald M. Schneeweiss

**Affiliations:** Department of Botany and Biodiversity Research, University of Vienna, Vienna, Austria

**Keywords:** bioinformatic pipeline, marker development, Orobanchaceae, phylogenomics, single copy nuclear genes, target enrichment

## Abstract

Phylogenomic approaches, employing next-generation sequencing (NGS) techniques, have revolutionized systematic and evolutionary biology. Target enrichment is an efficient and cost-effective method in phylogenomics and is becoming increasingly popular. Depending on availability and quality of reference data as well as on biological features of the study system, (semi-)automated identification of suitable markers will require specific bioinformatic pipelines. Here, we established a highly flexible bioinformatic pipeline, BaitsFinder, to identify putative orthologous single copy genes (SCGs) and to construct bait sequences in a single workflow. Additionally, this pipeline has been constructed to be able to cope with challenging data sets, such as the nutritionally heterogeneous plant family Orobanchaceae. To this end, we used transcriptome data of differing quality available for four Orobanchaceae species and, as reference, SCG data from monkeyflower (*Erythranthe guttata*, syn. *Mimulus g.*; 1,915 genes) and tomato (*Solanum lycopersicum*; 391 genes). Depending on whether gaps were permitted in initial blast searches of the four Orobanchaceae species against the reference, our pipeline identified 1,307 and 981 SCGs with average length of 994 bp and 775 bp, respectively. Automated bait sequence construction (using 2× tiling) resulted in 38,170 and 21,856 bait sequences, respectively. In comparison to the recently published MarkerMiner 1.0 pipeline BaitsFinder identified about 1.6 times as many SCGs (of at least 900 bp length). Skipping steps specific to analyses of Orobanchaceae, BaitsFinder was successfully used in a group of non-parasitic plants (three Asteraceae species and, as reference, SCG data from *Arabidopsis thaliana* based on previously compiled SCGs). Thus, BaitsFinder is expected to be broadly applicable in groups, where only transcriptomes or partial genome data of differing quality are available.

## Introduction

Combining target enrichment with next-generation sequencing (NGS) strategies can yield a large number of low copy nuclear (LCN) loci and is becoming increasingly popular for systematic and evolutionary biology (Lemmon and Lemmon, 2013). Target enrichment or sequence capture is a DNA-based method that uses hybridization of sheared DNA from the species of interest to conserved oligonucleotides (Zimmer and Wen, 2015). As it can also be applied to samples with already fragmented DNA as obtained from herbarium specimens, utilizing the wealth of material available in natural history collections becomes feasible (Paijmans et al., 2016; Suchan et al., 2016). Target enrichment relies on the availability of reference data, which may come from draft genomes, transcriptome data (RNASeq or EST data), genome skimming, or a combination thereof (Mandel et al., 2014; Weitemier et al., 2014; Schmickl et al., 2015). For example, Weitemier et al. (2014) and de Sousa et al. (2014) used whole genome data to identify 768 genes (≥960 bp) in milkweeds (*Asclepias*) and 50 genes (≥2,000 bp) in burclover (*Medicago*), respectively. Schmickl et al. (2015) combined genome skimming data from one sorrel species (*Oxalis obtusa*) with transcriptome data from another species (*Oxalis corniculata*) for targeting LCN loci. To this end, the authors implemented an automated and interactive bash script workflow for discovery of LCN loci for phylogenetic analysis. Chamala et al. (2015) and Mayer et al. (2016) developed MarkerMiner 1.0 and BaitFisher, respectively. Both tools provide an automated, user-friendly and web-supported workflow for discovery of LCN loci from transcriptome data. Campana (2017) developed BaitsTools, which automates bait design from various sources (e.g., alignments, unaligned sequences, and RADseq loci) and quality checking of thus obtained baits. Disadvantages of these approaches include reliance on whole or draft genome sequences (de Sousa et al., 2014; Weitemier et al., 2014) or settings that bias against less conserved loci, i.e., strictly reciprocal blast searches in MarkerMiner (Chamala et al., 2015) and the highly reduced bait-to-target distances in BaitFisher (Mayer et al., 2016). Additionally, none of these methods assessed the effect of using different blast strategies (with and without gaps) on number and length of recovered LCN loci.

The plant family Orobanchaceae encompasses a wide range of nutritional modes from non-parasitic autotrophy via photosynthetic parasitism to non-photosynthetic parasitism and contains some major pest species on economically important crop plants (Heide-Jørgensen, 2008). Orobanchaceae have become a model system for studying the evolution of parasitic plants, including molecular evolution under altered functional constraints (dePamphilis and Palmer, 1990; Wolfe and dePamphilis, 1998; Young and dePamphilis, 2005; Westwood et al., 2010; Wicke et al., 2013, 2016; Fan et al., 2016). Following these research avenues requires a solid phylogenetic framework, but although considerable progress has been made in reconstructing phylogenetic relationships within Orobanchaceae (Wolfe and dePamphilis, 1998; Young et al., 1999; Young and dePamphilis, 2005; Bennett and Mathews, 2006; Park et al., 2008; McNeal et al., 2013; Li et al., 2017), numerous areas of unknown or uncertain relationships remain (Schneeweiss, 2013). Phylogenomic approaches, although no panacea for every phylogenetic problem (Posada, 2016 and references therein), are needed to resolve phylogenetic relationships, but the identification of suitable genomic markers requires specific bioinformatic pipelines. Here, we establish a highly flexible bioinformatic pipeline, BaitsFinder, to discover putative orthologous single copy genes (SCGs) from Orobanchaceae and to construct bait sequences. To this end, we used transcriptome data of differing quality available from four Orobanchaceae species and SCG data of monkeyflower (*Erythranthe guttata* [syn: *Mimulus g.*], Phrymaceae) and tomato (*Solanum lycopersicum*, Solanaceae) as reference dataset. The Orobanchaceae data set can be considered challenging due to both biological characteristics (e.g., contamination by the parasites’ host species) and technical issues (transcriptome data of differing quality), and therefore should readily work in less complicated data sets. To test the general suitability of our pipeline, we assessed its performance using a data set from a non-parasitic group, the Asteraceae, which have been used in an early target-enrichment study in plants (Mandel et al., 2014).

## Materials and Methods

**FIGURE 1:**
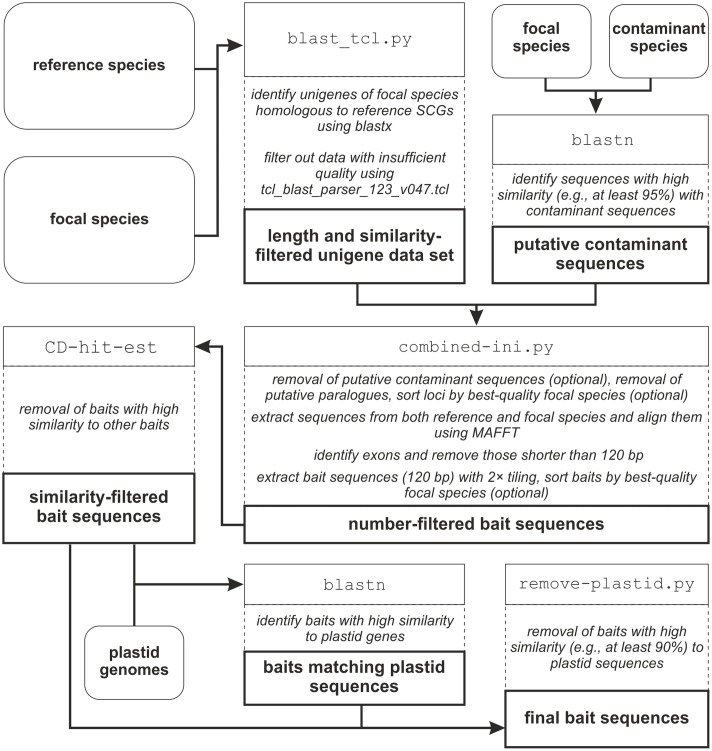
Workflow of the pipeline. External data are indicated by rounded boxes, data generated within the pipeline by thick-outlined boxes; programs and/or scripts are indicated by Courier New font in thin-outlined boxes; the rationale of each step is given in dashed out-lined boxes.

The workflow of BaitsFinder, which uses publicly available software and tools as well as several python scripts, is shown in **Figure 1**. All scripts were written in Python 2.7.9 and are available on GitHub^1^; the packages NumPy and SciPy^2^ need to be installed to run the python scripts. BaitsFinder has been tested on Linux (Ubuntu 16.04) and on Windows 7. A detailed description of all steps can be found in the step-by-step guide available on GitHub^1^. All blast searches used BLAST 2.2.6, as the output of BLAST+ is not compatible with tcl_blast_parser_123_V047.tcl used for some steps. Note that any of the used parameters (e.g., blast parameters, minimum overlap for identifying putative paralogues, or filtering parameters) may have to be adjusted for other study groups to ensure sufficient data quality and desired number of SCGs and bait sequences.

The first step is to identify SCGs in species that are closely related to our group of interest (here Orobanchaceae) and for which well-annotated genomes are available. We chose to use *E. guttata* (available as *Mimulus guttatus*)^3^ from Phrymaceae, a family phylogenetically close to Orobanchaceae and from the same order Lamiales, as primary reference (Hellsten et al., 2013) and the more distantly related, but better annotated *S. lycopersicum*^4^ from Solanaceae in the order Solanales, as secondary reference species (Fernandez-Pozo et al., 2015). The putative status as SCG was assessed by comparison against SCG data from *Arabidopsis thaliana* (compiled by Alexander Kozik and Richard Michelmore^5^) using blastx (Altschul et al., 1990). Briefly, *E. guttata* genes that had sufficiently high similarity to, but only a single match with *Arabidopsis* SCGs were identified and blasted against each other to retrieve genes that are single copy in *Erythranthe*. Using the same strategy, we obtained SCGs from *S. lycopersicum*. Finally, we merged the *Erythranthe* and *Solanum* SCG data sets, retaining only those SCGs from *Solanum* that had no significant blast hit with *Erythranthe* to avoid gene redundancy. The thus obtained reference data sets contained 2,306 SCG loci: 1,915 SCG sequences from *E. guttata*, our primary reference, and 391 SCG loci from *S. lycopersicum*, our secondary reference.

Data for the four Orobanchaceae species (our focal species) were downloaded from the Parasitic Plant Genome Project (PPGP) database^6^ (Yang et al., 2015): *Lindenbergia philippensis* (LiPhGnB1.fasta), *Triphysaria versicolor* (TrVeBC2.fasta), *Striga hermonthica* (StHeBC2.fasta), *Phelipanche (Orobanche) aegyptiaca* (OrAeBC4.fasta). These were blasted (with an *e*-value of 1*e*-10) against the reference database of non-redundant SCGs from *E. guttata* and *S. lycopersicum* SCGs. As across broader phylogenetic depths homologous genes might differ in length, we used both ungapped blastx (as done by Mandel et al., 2014) as well as gapped blastx searches. Subsequently, we parsed the blast output using tcl_blast_parser_123_V047.tcl^7^ and retained as putative single-copy conserved orthologous sequence (COS) the best query hit with identity of at least 40, expectation better than 1*e*-20, and an alignment length of at least 100 positions (on the amino acid level). The minimum length of 100 amino acid positions (i.e., 300 bp) is shorter than the length cut-off of 960 bp used by Weitemier et al. (2014), who used draft genome and transcriptome data from a congeneric reference species, but longer than that of 150 bp applied by Mandel et al. (2014). The combined builds from the parasitic species available from PPGP (all but *Lindenbergia*) may include sequences from their host species, which have to be removed prior to further processing. To this end, we blasted the Orobanchaceae COS sequences against sequences from their putative hosts using blastn and removed those sequences with identity of at least 95% to the host. Host sequences might be from species the parasite has actually been grown on (e.g., *P. aegyptiaca* has been grown on tobacco and *Arabidopsis*) or from close relatives. While this step is specific for parasitic species, it may be applied to non-parasitic species if contamination might be an issue. If not needed, this step can be skipped altogether.

In contrast to the approach used by MarkerMiner (Chamala et al., 2015), where putative paralogs are retained, we aimed at removing those as completely as possible. Different unigenes from the same focal taxon that have been blasted against the same reference SCG and that exceed a user-defined overlap cut-off are considered putative paralogues and are removed. The stringency of this approach will be determined both by data quality (e.g., many small unigenes versus fewer longer unigenes) and by the desired number of loci to create baits from.

Sequences from the focal species that have been blasted to the same gene from the reference species were extracted and put together in one folder, named after the reference protein ID. As the quality of the transcriptome data from the four Orobanchaceae species differs considerably, we retained only those loci that contained at least one sequence of *L. philippensis*, which based on the N50 value has the best assembled data among our focal species. Sorting by a best-quality focal taxon is optional and should be skipped if not needed, as filtering based on a single taxon can introduce a selection bias that may be propagated in case the selected genes are further subsampled based on the amount of variation across taxa. Alignment of each SCG was done using MAFFT 7.245 (Katoh et al., 2002) using the E-INS-i method, which is suitable for data sets containing multiple conserved domains and long gaps.

Each aligned SCG was split into smaller alignments each corresponding to an exon, whose boundaries in the genes from the reference species (*Erythranthe* and *Solanum*) have been extracted from the gff3 files (Tomato Genome Consortium, 2012; Hellsten et al., 2013) available from Phytozome 10.3^8^. We have no information on exon-intron boundaries in the four Orobanchaceae species, but assume that gene structure is sufficiently similar to that of *Erythranthe* and *Solanum*. If exon–intron boundaries are known for the group of interest, then separating exons should be done before the alignment. Thus obtained exon sequences were degapped, and exons < 120 bp (i.e., the length of the bait sequence) were removed. Probes (bait sequences) were designed automatically using a length of 120 bp and 2× tiling (i.e., baits overlap by 50%), allowing a maximum overlap of baits of 80 bp (i.e., from a fragment of 160 bp still two baits, overlapping by 80 bp, can be extracted). This threshold was chosen to make better use of the 3′-end of an exon while avoiding unduly high redundancy between baits. Loci that yielded fewer than four baits in *Lindenbergia* (i.e., the best assembled focal species) were removed, retaining loci that had at least 280 bp. To reduce redundancy among baits, probes that shared at least 90% identities among the four species were removed using cd-hit-est^9^ (Li and Godzik, 2006). Finally, in order to reduce the chance of targeting plastid genomes, loci containing at least one bait that in a blastn search had at least 90% identity with any of the 12 published plastid genomes from Orobanchaceae (Wicke et al., 2013) were removed as well. Recovery of, full or partial, plastid genomes may, however, still be possible by (reference-based) assembly of off-target reads (Weitemier et al., 2014). The same procedure may be used to remove baits potentially targeting mitochondrial genomes, if desired (no mitochondrial genome of Orobanchaceae published to data).

In order to test the general suitability of our pipeline, we additionally evaluated its performance in a group of non-parasitic plants. To this end, we used a data set of three species from the Asteraceae used previously for target-enrichment (Mandel et al., 2014): safflower (*Carthamus tinctorius*), sunflower (*Helianthus annuus*), and lettuce (*Lactuca sativa*;^10^ available from the Compositae Genome Project^11^). As reference data, we use *Arabidopsis* SCG data (following the annotation from Phytozome 10^12^) based on the 3,714 SCGs compiled previously (by Alexander Kozik and Richard Michelmore^13^). Several steps applied for Orobanchaceae were not necessary for Asteraceae and were, therefore, skipped: creating the reference data set, removal of putative host sequences and sorting based on a focal species with sufficient data quality. For removal of baits likely targeting plastid regions, plastid genomes for the three Asteraceae species were downloaded from GenBank: *Carthamus tinctorius* (KX822074); *Helianthus annuus* (DQ383815); and *Lactuca sativa* (AP007232).

## Results and Discussion

### Gapped versus Ungapped Blast

**FIGURE 2:**
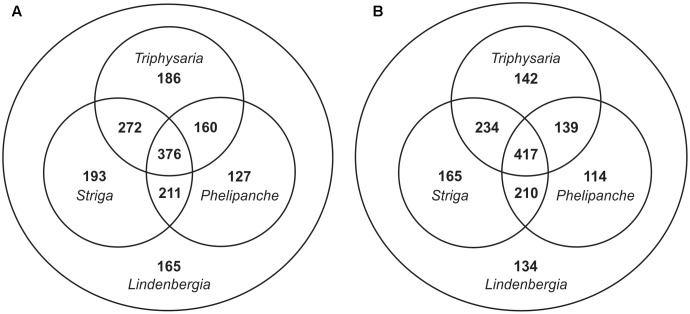
Distribution of SCGs among the four Orobanchaceae focal species recovered using **(A)** gapped blast or **(B)** ungapped blast (see text for details).

For identifying SCGs from the Orobanchaceae focal species, blastx allowing gaps (gapped blast) or not allowing gaps (ungapped blast) has been used. Except for some minor differences during the sequence alignment step, their results are processed equally. Filtering out baits likely targeting plastid genomes caused the removal of two loci with together 19 baits in case of gapped blast and of one locus with 7 baits in case of ungapped blast. As expected, using gapped blast resulted in more recovered loci: 2,050 SCGs in gapped blast versus 1,845 SCGs in ungapped blast and, after filtering for presence in *Lindenbergia*, 1,690 in gapped blast versus 1,555 in ungapped blast (a decrease of 17.6% and 15.7%, respectively; **Table 1**). Although gapped blast recovered more loci, the number of loci shared by all four focal species actually decreased while the number of loci shared by maximally three focal species increased as expected (**Figure 2**). The strategy using gapped blast recovered on average longer loci than the one using ungapped blast (**Table 1**). Therefore, the strategy using gapped blast yielded 38,170 baits (from 1,307 SCGs), which were 1.7 times more than the 21,856 baits (from 981 SCGs) obtained using the strategy with ungapped blast (**Table 1**).

**Table 1:** Number and characteristics of loci identified from the four Orobanchaceae species.

	SCGs		CDS		
Species data	Number	Average length	Number	Average length	Number of probes
**Gapped blast**					
LiPhGnB1	1,690	1,186	5,429	278	17,272
TrVeBC2	1,201	938	2,497	251	6,612
StHeBC2	1,278	985	2,878	264	8,510
OrAeBC4	1,055	868	2,095	259	5,776
**Ungapped blast**					
LiPhGnB1	1,555	846	3,934	228	9,312
TrVeBC2	1,079	754	2,094	220	3,912
StHeBC2	1,214	761	2,396	222	4,888
OrAeBC4	1,031	739	1,928	225	3,744

*Species data are builds of transcriptome data generated within the Parasitic Plant Genome Project (see http://ppgp.huck.psu.edu for details). The first four letters indicate the species: LiPh, *Lindenbergia philippensis;* TrVe, *Triphysaria versicolor;* StHe, *Striga hermonthica;* OrAe, Phelipanche (Orobanche) aegyptiaca*.

Similar trends were observed in Asteraceae (no plastid-targeting baits detected), where gapped blast yielded approximately 1.2× as many SCGs as ungapped blast (1,839 versus 1,528 SCGs, respectively). Likewise, the strategy using gapped blast recovered on average longer loci than the one using ungapped blast (**Table 2**), resulting in about 1.9 times more baits (16,310 baits from 1,744 SCGs versus 8,750 baits from 1,343 SCGs, respectively; **Table 2**). Mandel et al. (2014), who used the same Asteraceae data with ungapped blast only, obtained 9,678 baits from 1,061 SCGs, i.e., more than we did. This likely is due to our used length cut-offs (i.e., loci have to be at least 300 bp long and exons have to be at least 120 bp long).

**Table 2:** Number and characteristics of loci identified from the three Asteraceae species.

	SCGs		CDS		
Species data	Number	Average length	Number	Average length	Number of probes
**Gapped blast**					
*C. tinctorius*	1,062	663	5,435	278	5,236
*H. annuus*	866	638	2,503	251	4,083
*L. sativa*	1,232	710	2,880	264	6,991
**Ungapped blast**					
*C. tinctorius*	888	536	1,354	203	2,966
*H. annuus*	695	529	1,045	203	2,226
*L. sativa*	1,027	553	1,659	209	3,558

*Species data are assembled transcriptome data generated within the Compositae Genome Project (see http://cgpdb.ucdavis.edu/ for details)*.

The choice of blast strategy will depend on the divergence of the studied taxa as well as the desired number of bait sequences. For distantly related taxa (genus-level or above), where indels in alignments of coding sequences are more likely, gapped blast may be necessary to achieve the required number of loci and bait sequences. Ungapped blast, which gives more conservative results both with respect to number and length of recovered loci, may be the preferred choice for closely related taxa (i.e., from the intrageneric to intraspecific level) that may have a history of hybridization or introgression.

### Test of MarkerMiner 1.0 with the Orobanchaceae Dataset

We also employed the MarkerMiner 1.0 pipeline (Chamala et al., 2015) to compare its efficacy for developing SCGs with our pipeline using the Orobanchaceae data set. As data quality differs among our focal species, we only considered a single focal species, *L. philippensis*, which has the best quality data (i.e., the longest unigenes). As neither *Erythranthe* nor *Solanum* are included in the reference options available in MarkerMiner 1.0, we created a new reference, congruent with the one used as subject database in our pipeline (*E. guttata* and *S. lycopersicum* SCGs). We ran MarkerMiner 1.0 under the default settings, except for changing the reference. Consistent with MarkerMiner 1.0, which uses gapped blast for reciprocal blast searching, for comparison from our pipeline only the results from the gapped blast strategy were considered.

**FIGURE 3:**
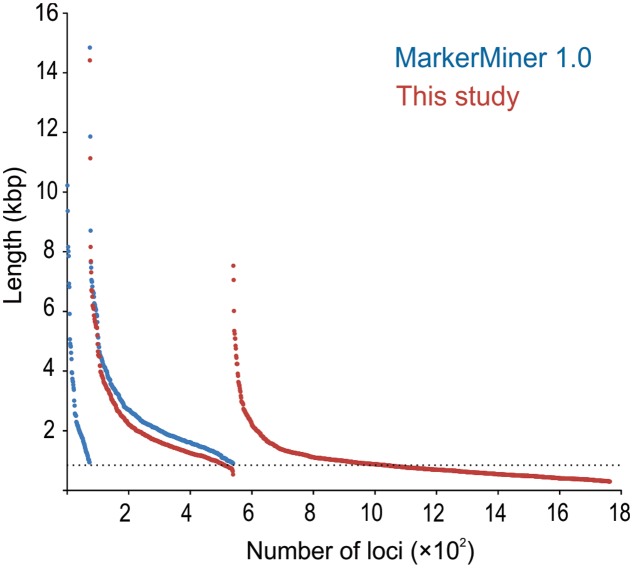
Length distribution of SCG loci of *Lindenbergia philippensis* identified by MarkerMiner (blue) and by BaitsFinder (red). Loci are organized in three panels (found by MarkerMiner only; found by both MarkerMiner and BaitsFinder; found by BaitsFinder only) and, within each panel, are sorted by length.

MarkerMiner 1.0 resulted in 539 loci (≥900 bp) compared to 865 loci (≥900 bp) recovered by our pipeline, of which 428 loci (≥900 bp) were shared by both; the number of shared loci increased to 466, if all 1,690 loci identified by our pipeline (i.e., also including those <900 bp) were considered (**Figure 3**). MarkerMiner identified 73 loci (by design ≥900 bp) not recovered by our pipeline. The reduced number of identified loci from MarkerMiner 1.0 likely is due to the default stringency criteria. MarkerMiner employs reciprocal blast, with both tblastn and blastx, while our pipeline uses only blastx. Secondly, in MarkerMiner the default length cut-off is 900 bp (compared to 280 bp in our study) and at least 70% of the query has to be aligned with 70% identity to the subject (compared to no overlap length requirements and 40% identity in our pipeline). Indeed, if the criteria in MarkerMiner 1.0 are relaxed (at least 40% of the query has to be aligned with 40% identity to the subject) in the reciprocal blast, the number of recovered loci increases to 1,466 loci. The reason for loci exclusively identified by MarkerMiner might be that this program retains loci with multiple hits from the same focal species, while in our pipeline such a locus would be removed from the focal species (not necessarily from the entire data set). One advantage of our method over MarkerMiner may be that it is very flexible in dealing with data, because it is a semi-automated pipeline consisting of several independent custom python scripts that can be easily modified to meet the demands of different studies. Another advantage of our method may be that it is stricter in removing likely paralogues, which will be helpful in targeting orthologous SCGs.

### General Comparison with Available Pipelines

There are also other pipelines available (**Table 3**). A major limitation is that both draft genomes (de Sousa et al., 2014; Weitemier et al., 2014) and genome skimming data (Schmickl et al., 2015) are still infrequent, and none of them is available for Orobanchaceae. Another limitation lies in the stringent criteria used by those pipelines (Mandel et al., 2014, 2015; Chamala et al., 2015; Mayer et al., 2016), which may result in losing a considerable amount of putatively informative sites by non-recovery of a large number of loci and by reduced length of loci (by not allowing gaps). This might negatively affect species tree estimation, whose accuracy has been found to be positively correlated with the number of putative SCGs, even in the presence of both high incomplete lineage sorting and gene rate heterogeneity (Xi et al., 2016). Additionally, these approaches enrich conserved loci, which may negatively affect the power to resolve phylogenetic relationships especially at the species level.

**Table 3:** Comparison of major pipelines.

Pipeline	Pros	Cons
MarkerMiner	• Equipped with reference database of orthologous genes	• May be biased toward conserved loci
	• Easy to use	• Paralogues are not removed
		• Does not generate bait sequences
BaitFisher	• Yields high quality baits	• Susceptible to low-quality data
		• Number of utilized loci may be low
BaitsTools	• Allows variant selection, bait generation, and bait quality control	• Does not include (internal or external) tools to modify or generate input data (e.g., produce alignments, identify single copy genes)
	• Supports a variety of input formats	
BaitsFinder	• Flexible and easy to modify	• Limited bait quality check
	• Takes a number of potential complications into account (e.g., contaminations, data of heterogeneous quality)	

In contrast to our pipeline, MarkerMiner (Chamala et al., 2015) outputs SCG sequences, but not bait sequences. MarkerMiner may, however, be combined with other tools such as BaitFisher (Mayer et al., 2016) or BaitsTools (Campana, 2017). BaitFisher has been developed to obtain an optimal set of baits by minimizing the number of baits (by reducing redundancy of baits without gaps or ambiguous nucleotides) while maximizing the number of targeted nucleotide sequences. The disadvantage of BaitFisher is that it does not tolerate any gaps nor ambiguity codes in the start position of a putative bait, rendering it susceptible to low-quality samples. BaitsTools (Campana, 2017) can use various forms of input data (e.g., alignments, sequence lists, RADseq loci) and allows quality checks on obtained bait sequences to be performed, but it does not include any tools to modify or generate input data (e.g., removal of putative contaminants, extracting exons from transcriptome data).

## Conclusion

We established a highly efficient bioinformatic pipeline, BaitsFinder, that employs blast searches, alignment, length-filtering, and sorting to discover putative orthologous SCGs from transcriptome data. Although no (draft) whole genome sequence is available for our focal group, the Orobanchaceae, we were able to develop up to 38,170 baits (representing 1,307 loci) from combined transcriptomes of four Orobanchaceae species. A comparison of BaitsFinder with MarkerMiner 1.0 suggests that our pipeline recovers 1.6 times as many loci as MarkerMiner (used with its default settings), which ultimately may help in improving the accuracy of species tree estimation. As BaitsFinder is very flexible and takes into account a number of complications (contamination by, for instance, host sequences, low quality reference data), it should be readily applicable to other non-model plants as shown here for Asteraceae.

## Availability of Data and Materials

Python scripts and example data are available from GitHub: https://github.com/plantbiogeography/BaitsFinder. Reference data for Orobanchaceae (SCGs of *Erythranthe* and *Solanum*) are available at Dryad under doi: 10.5061/dryad.7b86c.

## Author Contributions

GS and XL designed the pipeline. BH wrote the python scripts. DP and XL tested the pipeline. XL and BH analyzed the data. GS and XL prepared the manuscript. All authors read and approved the final manuscript.

## Conflict of Interest Statement

The authors declare that the research was conducted in the absence of any commercial or financial relationships that could be construed as a potential conflict of interest.

## Footnotes

1 https://github.com/plantbiogeography/BaitsFinder

2 https://www.scipy.org/\#

3 https://phytozome.jgi.doe.gov/pz/portal.html

4 ftp://ftp.solgenomics.net/genomes/Solanum_lycopersicum/annotation/ITAG2.4_release/

5 http://cgpdb.ucdavis.edu/COS_Arabidopsis/data/arabidopsis_single_copy_genes.fasta

6 http://ppgp.huck.psu.edu/download.php

7 http://code.google.com/p/atgc-tools/downloads/detail?name=tcl_blast_parser_123_V047.tcl

8 https://phytozome.jgi.doe.gov/pz/portal.html

9 http://weizhong-lab.ucsd.edu/cdhit_suite/cgi-bin/index.cgi

10 http://cgpdb.ucdavis.edu/asteraceae_assembly/

11 http://cgpdb.ucdavis.edu/

12 https://phytozome.jgi.doe.gov/pz/portal.html

13 http://cgpdb.ucdavis.edu/COS_Arabidopsis/data/arabidopsis_single_copy_genes.fasta
